# Pancreatoduodenectomy for Pancreatic Cancer after Prior Modified Puestow Procedure: A Case Report

**DOI:** 10.70352/scrj.cr.25-0821

**Published:** 2026-03-07

**Authors:** Yuiko Nagasawa, Teijiro Hirashita, Wataru Miyoshino, Shun Nakamura, Masahiro Kawamura, Hiroomi Takayama, Yoko Kawano, Takashi Masuda, Yuichi Endo, Masafumi Inomata

**Affiliations:** Department of Gastroenterological and Pediatric Surgery, Oita University Faculty of Medicine, Yuhu, Oita, Japan

**Keywords:** chronic pancreatitis, Puestow procedure, intraductal papillary mucinous carcinoma, pancreatoduodenectomy, pancreatic cancer after chronic pancreatitis

## Abstract

**INTRODUCTION:**

Chronic pancreatitis is a known risk factor for pancreatic cancer and may develop over the course of long-term disease management. When a pancreatectomy is required following pancreatic duct decompression surgery, careful consideration of the surgical approach is necessary due to altered anatomy. This study reports a case of pancreatoduodenectomy for intraductal papillary mucinous carcinoma after the modified Puestow procedure.

**CASE PRESENTATION:**

A 52-year-old man with chronic alcoholic pancreatitis underwent the modified Puestow procedure for pancreatic duct decompression following an unsuccessful endoscopic stone removal. Ten years later, the patient presented with obstructive jaundice. Imaging revealed a pancreatic head tumor with biliary dilatation and an adjacent pancreatic pseudocyst, confirming the diagnosis of pancreatic cancer. After neoadjuvant chemotherapy with gemcitabine plus S-1 and endoscopic management of the infected pseudocyst, pancreatoduodenectomy was performed. A pancreaticojejunostomy was performed utilizing the jejunal limb fashioned during the modified Puestow procedure thereby minimizing the need for additional bowel resection. Pathological examination revealed invasive intraductal papillary mucinous carcinoma. The postoperative course was uneventful, and no recurrence was observed at 36 months.

**CONCLUSIONS:**

Pancreatoduodenectomy for intraductal papillary mucinous carcinoma after the modified Puestow procedure can be safely performed with meticulous preoperative planning.

## Abbreviations

CA19-9
: carbohydrate antigen 19-9
CP
: chronic pancreatitis
EUS-FNA
: endoscopic ultrasound-guided fine needle aspiration
GS
: gemcitabine and S-1
IPMC
: intraductal papillary mucinous carcinoma
IPMN
: intraductal papillary mucinous neoplasm
LAMS
: lumen-apposing metal stent
PD
: pancreatoduodenectomy
PJ
: pancreaticojejunostomy
SIR
: standardized incidence ratio

## INTRODUCTION

Pancreatic cancer remains one of the most lethal malignancies worldwide, with a steadily increasing incidence. The age-standardized incidence rate is projected to exceed 15 per 100000 by 2030.^1)^ CP is a well-established risk factor for pancreatic ductal adenocarcinoma and may be complicated by pancreatic malignancy during long-term disease management. In contrast, a causal association between CP and IPMN has not been clearly established. When pancreatic resection becomes necessary after pancreatic duct decompression surgery for CP, substantial technical challenges may be encountered due to altered anatomy and severe adhesions. Herein, we report a case of PD performed for IPMC diagnosed during long-term follow-up after a modified Puestow procedure.

## CASE PRESENTATION

A 52-year-old man with no significant medical history reported long-term heavy alcohol consumption (approximately half a bottle of Japanese liquor daily) and a 40-year history of smoking. Pancreatic stones were detected incidentally on abdominal CT during a routine health checkup. After discontinuation of alcohol intake, the patient experienced significant weight loss accompanied by anorexia and general fatigue and was subsequently diagnosed with alcoholic CP with pancreatic stones at another hospital. Endoscopic procedures, including pancreatic stone removal via endoscopic retrograde cholangiopancreatography, were also attempted. However, during the procedure, the stone retrieval basket became irretrievably impacted within the main duct of the pancreatic head. The patient was urgently transferred to our institution, with the endoscopic basket remaining *in situ*. Given the presence of extensive pancreatic duct stones, predominantly in the pancreatic tail, and the need to address the retained intraductal basket, surgical intervention was considered the most appropriate treatment. Consequently, a modified Puestow procedure was performed, comprising resection of the pancreatic tail combined with a longitudinal PJ.

**Fig. 1:**
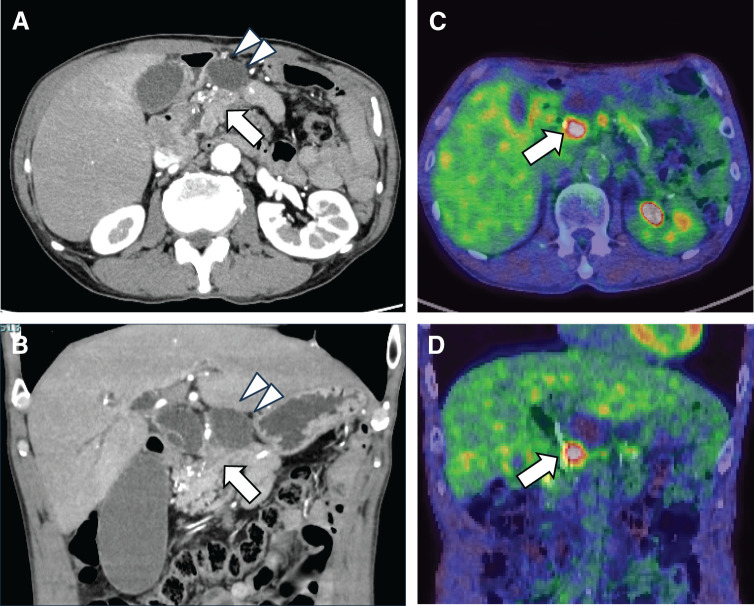
Preoperative imaging findings. Contrast-enhanced CT in the sagittal and coronal views (**A**, **B**) and PET-CT in the sagittal and coronal views (**C**, **D**) demonstrate a pancreatic head tumor (arrows) located adjacent to a pancreatic pseudocyst (arrowheads), accompanied by dilatation of the intrahepatic bile ducts. Increased fluorodeoxyglucose uptake is observed in the pancreatic head tumor.

Ten years post-modified Puestow procedure, the patient was referred to our hospital with progressive jaundice. One year before presentation, a cystic lesion in the pancreatic head was detected and followed up conservatively. He had a concomitant diagnosis of type 2 diabetes mellitus. A physical examination revealed scleral and cutaneous jaundice. His abdomen was flat and soft, without tenderness. Laboratory findings revealed mild hyperbilirubinemia with elevated hepatobiliary enzymes: total bilirubin 5.87 mg/dL, direct bilirubin 4.55 mg/dL, aspartate aminotransferase 457.1 IU/L, alanine aminotransferase 174.4 IU/L, and γ-glutamyl transpeptidase 1931.3 IU/L. However, serum amylase level was within the normal range (46 U/L). Tumor marker analysis revealed an elevated CA19-9 at 253.3 U/mL. Contrast-enhanced CT revealed a hypovascular tumor measuring approximately 2 cm in size in the pancreatic head, accompanied by dilatation of the intrahepatic and extrahepatic bile ducts. A large pancreatic pseudocyst was observed adjacent to the tumor (**Fig. 1A**, **1B**). PET-CT demonstrated increased fluorodeoxyglucose uptake in the pancreatic head lesion, consistent with malignancy (maximum standardized uptake value, 4.6 on early-phase imaging, and 7.2 on delayed-phase imaging) (**Fig. 1C**, **1D**). EUS-FNA performed twice (4 needle passes) revealed atypical epithelial cells positive for p53 and Ki-67, suspicious for malignancy, although a definitive diagnosis could not be established. Based on these findings, the patient was diagnosed with pancreatic head cancer.

**Fig. 2:**
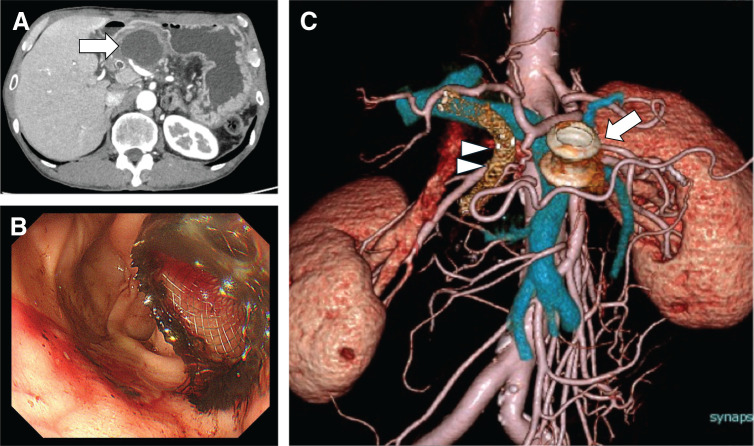
Endoscopic management and preoperative anatomical assessment. **(A)** The pancreatic pseudocyst progressed to infectious pancreatic necrosis during chemotherapy. **(B)** Endoscopic ultrasound-guided transgastric drainage using a lumen-apposing metal stent. **(C)** Reconstructed CT image demonstrates vascular anatomy and the location of the biliary and lumen-apposing metal stents.

Endoscopic biliary drainage using a metal stent was performed to relieve obstructive jaundice, followed by neoadjuvant chemotherapy with GS. During chemotherapy, the pancreatic pseudocyst progressed to infectious pancreatic necrosis, prompting endoscopic ultrasound-guided transgastric drainage with placement of a LAMS (**Fig. 2**). After 2 courses of chemotherapy, radiological evaluation indicated stable disease, with the serum CA19-9 levels decreasing to 32.21 U/mL.

**Fig. 3:**
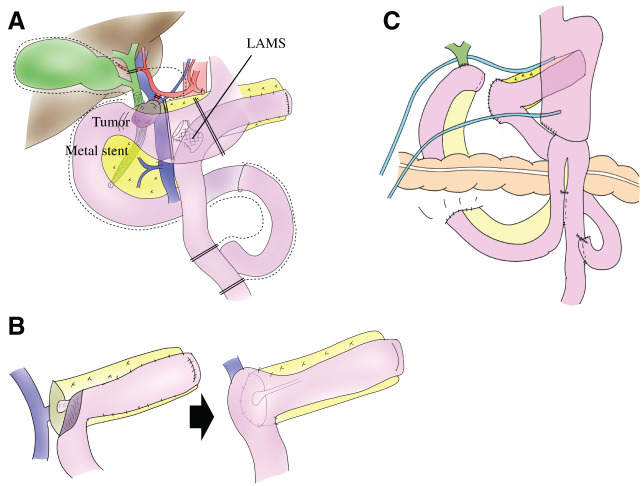
Schematic illustration of intraoperative findings and reconstructive strategy after modified Puestow procedure. This schematic illustration depicts the key intraoperative findings and reconstructive steps in the absence of intraoperative photographs. (**A**) Overview of the operative field. Severe adhesions and altered anatomy resulting from the previous modified Puestow procedure are observed. The jejunal limb used for the prior pancreaticojejunostomy is identified and preserved. The lumen-apposing metal stent placed for pancreatic pseudocyst drainage is identified within the jejunal limb and retrieved by partial division of the jejunum. (**B**) The jejunal orifice created by stent removal is utilized to reconstruct the pancreaticojejunostomy using an invagination technique. (**C**) Overall view after reconstruction. LAMS, lumen-apposing metal stent

Subsequently, PD with lymphadenectomy was performed. Severe adhesions and fibrotic changes were observed around the pancreatic head and mesentery, reflecting the modified Puestow procedure and CP. The LAMS, initially placed transgastrically for pancreatic pseudocyst drainage, was later identified within the jejunal limb at the previous PJ site following cyst shrinkage (**Fig. 3A**). The stent was safely retrieved by careful division of the involved jejunal segment, while preserving the remaining jejunal limb (**Fig. 3B**). The pancreas was transected at the portal vein level, and no malignancy was identified at the pancreatic cut margin. Reconstruction was performed using the jejunal limb created during the previous modified Puestow procedure. The pancreatic stump was anastomosed to the jejunum in continuity with the existing anastomosis using an invagination technique, thereby minimizing additional bowel resection (**Fig. 3C**). The operative time was 535 minutes, with an estimated blood loss of 610 mL. No intraoperative complications were observed.

**Fig. 4:**
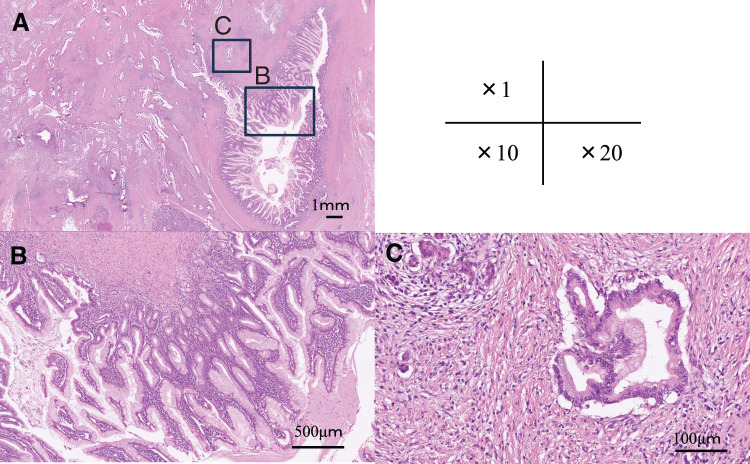
Histopathological findings of invasive intraductal papillary mucinous carcinoma (IPMC). (**A**) Low-power overview shows marked dilatation of the pancreatic duct with intraductal papillary proliferation of mucin-producing epithelial cells and irregularly shaped neoplastic glands infiltrating beyond the ductal wall into the surrounding fibrotic stroma (hematoxylin and eosin staining, ×1). (**B**) Intermediate magnification demonstrates intraductal papillary growth of mucin-producing epithelial cells within the dilated pancreatic duct (hematoxylin and eosin staining, ×10). (**C**) High-power view highlights irregular neoplastic glands infiltrating the fibrotic stroma outside the ductal wall, confirming the presence of an invasive carcinoma component (hematoxylin and eosin staining, ×20).

Histologically, papillary neoplastic lesions with complex branching architecture were observed within the dilated main pancreatic duct and branch ducts, composed of tumor cells with enlarged oval-to-round nuclei with prominent nucleoli and goblet cells with pale eosinophilic cytoplasm. In addition, irregularly shaped neoplastic glands were identified infiltrating beyond the ductal wall into the surrounding fibrotic pancreatic stroma, accompanied by a desmoplastic reaction. These findings were consistent with a diagnosis of invasive IPMC (**Fig. 4**). Pathological examination revealed a final stage of ypT3N0M0, corresponding to Stage IIa, with a histological therapeutic effect graded as Ib. The postoperative course was uneventful, and the patient was discharged on POD 13. The patient received 6 months of adjuvant chemotherapy with S-1 and survived without any evidence of disease recurrence at 36 months after surgery.

## DISCUSSION

CP is a well-established risk factor for pancreatic ductal adenocarcinoma, driven by long-standing fibroinflammatory changes, ductal remodeling, and accumulated genetic alterations.^2)^ Tobacco and alcohol consumption further potentiate pancreatic carcinogenesis.^2)^ A major diagnostic challenge is that CP itself can mimic malignancy both clinically and radiologically; inflammatory strictures or pseudotumorous changes may obscure early cancer, underscoring the importance of careful longitudinal assessment and timely tissue acquisition when suspicious findings emerge.^2)^ In Japan, recent multicenter data have demonstrated a substantially increased cancer burden among patients with CP. The Japan Pancreatitis Study Group reported a markedly elevated standardized incidence ratio for pancreatic cancer (SIR 6.44), highlighting pancreatic malignancy as a major determinant of long-term prognosis in this population.^3)^ Sakorafas and Sarr reported pancreatic cancer in 14 of 484 surgically treated CP patients (2.9%), with a mean interval of 3.4 years from surgery to cancer diagnosis.^4)^ Nevertheless, routine surveillance for pancreatic cancer in all patients with CP remains controversial.

Recent epidemiological analyses have further indicated that CP confers a substantially elevated risk of pancreatic cancer, which persists even after excluding cancers diagnosed shortly after CP recognition, suggesting that CP-associated carcinogenesis extends beyond detection bias.^3,5)^ This observation is clinically relevant in the present case, in which a pancreatic head malignancy developed long after pancreatic duct decompression, reinforcing the need for continued vigilance even after symptom control.^2,3)^

Pancreatic duct decompression procedures, including lateral PJ (Puestow or related procedures), are effective for pain relief and reduction of ductal hypertension in CP. Some studies have suggested that surgical treatment for CP may reduce subsequent pancreatic cancer risk; nevertheless, pancreatic malignancy can still occur years or even decades after decompression.^6)^ Matsumoto et al. reported a resected case of pancreatic head cancer developing 40 years after lateral PJ, illustrating both the long-term oncologic uncertainty and the technical challenges associated with resection in patients with prior PJ.^7)^

Reports describing PD after a prior Puestow procedure remain scarce. Nellen et al. documented completion PD following a Puestow procedure in hereditary pancreatitis, demonstrating technical feasibility while underscoring the complexity of redoing pancreatic surgery.^8)^ These limited reports indicate that each additional well-documented case provides valuable insight into operative planning and reconstruction strategies under altered anatomy (**Table 1**). Several factors contribute to the technical difficulty of pancreatic resection after CP treatment, including dense adhesions from chronic inflammation and prior surgery, altered intestinal anatomy such as Roux-en-Y limbs and previous PJ, distortion of biliary and pancreatic ductal anatomy, and challenges in interpreting postoperative changes on imaging.^9)^ These factors may prolong operative time, increase the risk of enteric or vascular injury, and complicate reconstructive decision-making.

**Table 1:** Previously reported cases of pancreatic cancer in CP patients undergoing surgical treatment

Author	Year	Surgical Procedure for CP	Time to cancer	Cancer type	Surgical procedure for cancer
Nellen^8)^	2018	Modified Puestow	25 years	Adenocarcinoma	Limited DP, splenectomy, cholecystectomy
Matsumoto^7)^	2024	Lateral PJ	40 years	Invasive ductal carcinoma	PD
Our Case	2026	Modified Puestow	10 years	IPMC	PD

CP, chronic pancreatitis; DP, distal pancreatectomy; IPMC, intraductal papillary mucinous carcinoma; PD, pancreatoduodenectomy; PJ, pancreaticojejunostomy

Accordingly, safe PD after pancreatic duct decompression surgery requires meticulous preoperative evaluation of reconstructed bowel anatomy, anastomotic sites, and indwelling stents, as well as individualized reconstructive strategies tailored to the altered surgical field.^9)^ In the present case, detailed preoperative planning enabled preservation of the jejunal limb created during the prior modified Puestow procedure and utilization of the stent-extraction orifice for invagination PJ, thereby minimizing additional bowel resection and achieving a safe, oncologically adequate operation.

## CONCLUSIONS

In patients undergoing pancreatic resection after a prior modified Puestow procedure, careful preoperative planning is essential to ensure safe resection under altered anatomical conditions. Careful planning combined with tailored surgical approaches allows safe execution of PD without compromising oncological radicality.

## DECLARATIONS

### Availability of data and materials

The data that support the findings of this study are available from the corresponding author upon reasonable request.
